# Passive Observer of Activities for Aging in Place Using a Network of RGB-D Sensors

**DOI:** 10.1155/2020/8867926

**Published:** 2020-10-23

**Authors:** Shahram Payandeh, Jim Park

**Affiliations:** Network Robotics and Sensing Laboratory, School of Engineering Science, Simon Fraser University, 8888 University Drive, Burnaby, British Columbia, Canada

## Abstract

Aging in place is a notion which supports the independent living of older adults at their own place of residence for as long as possible. To support this alternative living which can be in contrast to various other types of assisted living options, modes of monitoring technology need to be explored and studied in order to determine a balance between the preservation of privacy and adequacy of sensed information for better estimation and visualization of movements and activities. In this paper, we explore such monitoring paradigm on how a network of RGB-D sensors can be utilized for this purpose. This type of sensor offers both visual and depth sensing modalities from the scene where the information can be fused and coded for better protection of privacy. For this purpose, we introduce the novel notion of passive observer. This observer is only triggered by detecting the absence of movements of older adults in the scene. This is accomplished by classifying and localizing objects in the monitoring scene from both before and after the detection of movements. A deep learning tool is utilized for visual classification of known objects in the physical scene followed by virtual reality reconstructing of the scene where the shape and location of objects are recreated. Such reconstruction can be used as a visual summary in order to identify objects which were handled by an older adult in-between observation. The simplified virtual scene can be used, for example, by caregivers or monitoring personnel in order to assist in detecting any anomalies. This virtual visualization can offer a high level of privacy protection without having any direct visual access to the monitoring scene. In addition, using the scene graph representation, an automatic decision-making tool is proposed where spatial relationships between the objects can be used to estimate the expected activities. The results of this paper are demonstrated through two case studies.

## 1. Introduction

Partly motivated by the preference of majority of abled older adults (55-80 years old) and partly due to the existence of the current pandemic (COVID-19) which initially has affected living communities of older adults, aging in place has been gaining increased popularity [1, 2]. However, in order to support this alternative living lifestyle, there also should exist acceptable technology which allows older adults sharing a wide range of data associated with their health and well-being [3, 4]. State-of-the art in sensing technology offers several alternatives which can be used for monitoring movements and activities. This can be, for example, various environmental type sensors which can be placed in the living space of older adults for their interaction with appliances in terms of electric, heat, or water flow sensing, e.g., see [5–7]. Wearable sensors can offer an alternative sensing for monitoring movements and further extrapolation of their activities from the recorded data [8]. Visual RGB sensing can also be used as a comprehensive sensing which can be used to extract relevant information [9]. More recently, depth sensors are also utilized for detection of human postures which can then be used as a part of the overall activity recognition, see [10, 11]. A combination of both RGB and depth sensors (RGB-D) is used as a rich sense of modality in estimating human movements and activities [12–14].

In general, increased level of detail in sensing information from the monitoring area depends on the type, graduality, and the distribution of the sensors. For example, the basic distance sensor can determine the presence and the absence of an older adult within certain proximity defined by the operation properties of the sensor. On the other hand, RGB sensor can be used to obtain increased amount of details from the scene including the presence of the older adult [15]. However, protection of privacy has increasingly become one of the main concerns among older adults. Concerns are with the deployment of sensing modalities with an increased level of sensing details such as RGB camera [16–18]. As such, it is required that for a successful deployment of any sensing technologies, a balance be reached between the monitoring requirements and protection of their privacy.

This paper explores such novel synthesis between the rich sensing modalities offered through RGB-D sensing and the notion of AI-enabled passive monitoring for increased level of privacy associated with the monitoring system. The proposed AI-based approach is based on the scene analysis associated with the before and after detecting the presence of older adult in the monitoring area. One of the applications of the proposed system is in the development of a playback module for cases when an older adult has forgotten the placement of some objects and can use the system to backtrack some part of their activities as a reminder. The method uses a deep learning tool for detecting the presence of known objects in the living environment using RGB sensors. Using a calibration model of depth sensors, the method then localizes and summarizes the spatial positions of the detected objects in the virtual description of the scene. By comparing the before and after locations of detected objects in the scene description, it is possible to identify whether an older adult has interacted with the objects or any have taken away or introduced in the scene. In addition, it is also possible to mark the virtual scene with visual marker that identifies the movement trajectory of older adult which can be further explored in relation to location of the objects. The virtual summary results of this paper can be used by the caregiving professional or family members who can periodically observed the visual graphical summary in order to access any onset of anomalies.

The paper is organized as follows: Section 2 presents an overview of some tools which are used as a part of the design and development of this paper. This includes a method for sensor calibration and a deep learning object classification (YOLO) proposed by Redmon et al. [19].  Section 3 presents an overview of our proposed novel passive monitoring system and virtual scene reconstruction.  Section 4 presents sample case studies associated with the proposed passive monitoring system.  Section 5 presents a method based on scene graph analysis which was used for inferring on whether various objects were handled by the older adult. The results of this section were used in the previous section in annotation of the virtual scenes, and finally, Section 6 presents concluding remarks.

## 2. Background Materials

This section presents an overview of the two main tools which were used as a part of the design and development of this paper. The framework of this paper is based on the usage of multiple sensors which can be distributed in the living space of an older adult. As such, a relationship needs to be established between the spatial locations of the objects located in the sense which can be described with respect to a common coordinate frame (i.e., world coordinate) and with respect to coordinate frame of each sensor. This step is referred to as the calibration procedure which establishes a relationship between the RGB-D sensor frames and a coordinate located in the physical scene. In addition, calibration information from the RGB sensors is used in connection with a deep learning algorithm (YOLO) for localization of detected objects. Information regarding the identified objects and the calibration paraments of the sensor network are then used to segment point clouds of depth sensors.

### 2.1. Sensor Network Calibration

There exist several approaches for calibrating a network of RGB-D sensors. Camera calibration is the process of estimating the parameters of imaging system (intrinsic parameters) and the relative poses of the coordinate frame of the sensor with respect to a coordinate frame located in the scene. Estimation of these parameters can then be utilized in measuring locations and sizes of objects in units used for their physical descriptions. Calibration parameters can also be used in determining the actual location of the camera in the scene and to correct for any lens distortions. For the case of a single RGB camera, calibration is a study on how 3D points in the space are projected onto 2D pixels array in the image plane. For example, Tsai [20] and Zhang [21] proposed a planar patterned object in order to calibrate a single camera in a small field of view (FoV). Other calibration objects such as spheres were used for better analysis of camera calibration such as conic extraction that was proposed by Lu and Payandeh [22, 23] and Wang and Payandeh [24].

**Figure 1:**
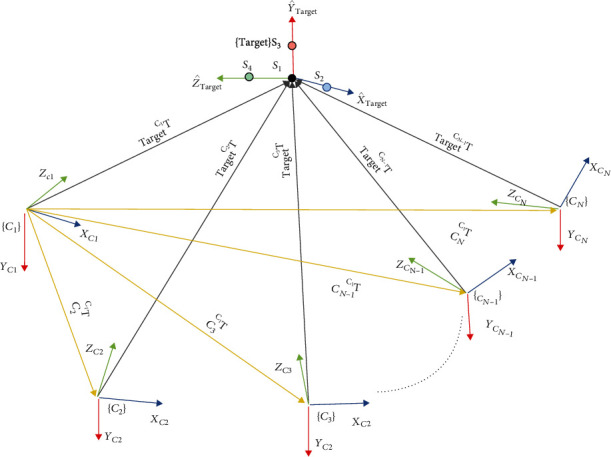
Definition of coordinate frame assignments and relative spatial descriptions of homogeneous frames in a network of multiple sensors. Here, arrows representing the relative descriptions between two frames (e.g., position and orientation of a frame that the head of the arrow is pointing with respect to the coordinate frame where the tail of the arrow is originating from).

A network of multiple sensors incorporates and fuses multiple sensing data from different viewpoints. This is accomplished by incorporating the extrinsic description of their relative spatial parameters obtained through multiple-sensor calibration. For instance, Krumm et al. [25] and Dai and Payandeh [26] demonstrated on how a single or multiple object can be labeled and tracked across multiple RGB surveillance cameras. Using two cameras, Wang and Payandeh [24] studied hand motion and posture recognition, and Gao et al. [27] investigated a full body motion capture. In order to integrate data from multiple sensors, it is required that both their intrinsic parameters and their relative locations (i.e., extrinsic parameters) be determined in order to increase accuracy and correspondences between cameras [23]. For example, Figure 1 shows a typical assignment of reference frame to multiple sensors which is designated by {*C*_*i*_} and a reference frame representing a target object (e.g., calibration prop/marker) designated by {Target}. In Figure 1, an arrow which points from the origin of a reference frame to another frame represents a correspondence to a homogeneous representation describing relative position and orientation of one frame (where the head of the arrow is pointing) with respect to another frame (where the tail of the arrow originating). Each of the arrows marked with _Target_^*C*_*i*_^*T* represents a homogeneous transformation (extrinsic parameter) between the *Target* frame where the measurements of the physical objects are obtained and the reference coordinate frame of each sensor. The result of calibration is used to compute the relative transformation between each of the sensors, e.g., _*C*_3__^*C*_1_^*T*, which is the relative description of the reference frame of sensor 3 with respect to the reference coordinate of the sensor 1. Such description can be used to combine the description of the objects from each sensor into a common coordinate frame.

**Figure 2:**
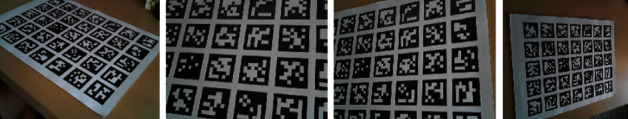
Different viewpoints of a ChArUco calibration prop/object. Depending on the location and configuration of each sensor with respect to the monitoring area, one needs to ensure that each sensor has an unambiguous view of the calibration prop. For all the experiments associated with this paper, the physical size of the marker is adjusted, and its placement is configured with respect to the common viewing angles of all of the sensors in order to ensure accurate calibration results. The figure shows four typical views of the common calibration object with respect to the sensor.

In this paper, we utilized OpenCV calibration tool based on ArUco markers and ChArUco board [28]. This function establishes correspondences between the objects in the environment and corners of calibration chart (prop/object). Calibrating using ArUco is much more versatile than using traditional checkerboard patterns, since it allows occlusions or partial views of the chart. Detection of the calibration prop/object also defines a reference coordinate system of the monitoring scene (i.e., world coordinates) to which point clouds of all the depth sensors are merged. Detection of the calibration prop/object (as shown in the example of Figure 2) is the first step in the analysis of the propose method before initiating further detection and classification of objects.

As shown in the Figure 2, from perspective of each sensor, the viewing angles of the calibration prop/object are different. From each camera angle, the computed information of where the common world coordinate reference frame can be established. This allows the association of point cloud of each sensor in order to be reconstructed in common frame of reference.

### 2.2. Object Detection

In recent years, many deep learning object detection tools have been proposed which promise to offer a robust detection framework [19]. In this paper, we proposed to explore how such tools can be utilized in the context of the passive observer of a monitoring area. As such, no attention was given to the speed of detection or any other requirements which may be associated with the general object detection problems. For example, two of the fastest detection algorithms are EfficientDet [29] and Yolo [19]. As a proof of concept of the proposed integrated system of this paper, we have selected YOLO for our implementation. In the following, we present some basic overview of this algorithm.

You Only Look Once [19] is one of the fast object detection methods which presents the detection as a regression problem to spatially separated bounding boxes and to associate class probabilities. A single neural network predicts bounding boxes and class probabilities directly from full images in one evaluation. YOLO sees the entire image during training and test time, so it implicitly encodes contextual information about classes as well as their appearance. The system splits the input image into *S* × *S* grid. If the center of an object falls into a grid cell, that grid cell is responsible for detecting that object. Within the grid, it takes *m* bounding boxes. For each of the bounding boxes, the network outputs a class probability. The bounding boxes having the class probability above a threshold value are selected and used to locate the object within the image.

Each grid cell predicts *B* bounding boxes and confidence scores for those boxes. These scores reflect how confident the model is that the box contains an object and how accurate it thinks the box is that it predicts. To each bounding box, it can be associated a prediction set such as the coordinate of the center of the box and its width and height, or *x*, *y*, *w*, *h*, and confidence coefficient. The (*x*, *y*) coordinates represent the center of the box relative to the bounds of the grid cell. The width and height are predicted relative to the whole image. This vector can be extended to include other parameters which can be used as a part of the detection process. To train the neural networks, the input image is used in a single convolution neural network with multiple convolution and Max pool layers. This neural network maps the input image to a smaller size feature vector. This involves matching the center of detected object to the correct cell and bounding boxes and its associated coordinates. The method uses both forward and backward propagation to train the model.

The method also has its own approach of encoding bounding boxes. *b*_*x*_, *b*_*y*_, *b*_*h*_, and *b*_*w*_ are calculated relative to its associated grid cell. Assuming the midpoint of the object is detected, its coordinates *b*_*x*_ and *b*_*y*_ are with respect to the upper left corner of the grid. *b*_*h*_ and *b*_*w*_ are ratio of the height and width of the bounding box to the height and width of corresponding grid cell. In case there are multiple objects in a single grid, YOLO implements the notion of anchor boxes. They are predefined shapes where each grid can have more than one output for its bounding boxes. For testing, the new image will be divided into the same number of grids which we have chosen during the training period. For each grid, the model will predict an output of shape (assuming this is the shape of the target during training time).

## 3. Overview of the Passive Observer

**Figure 3:**
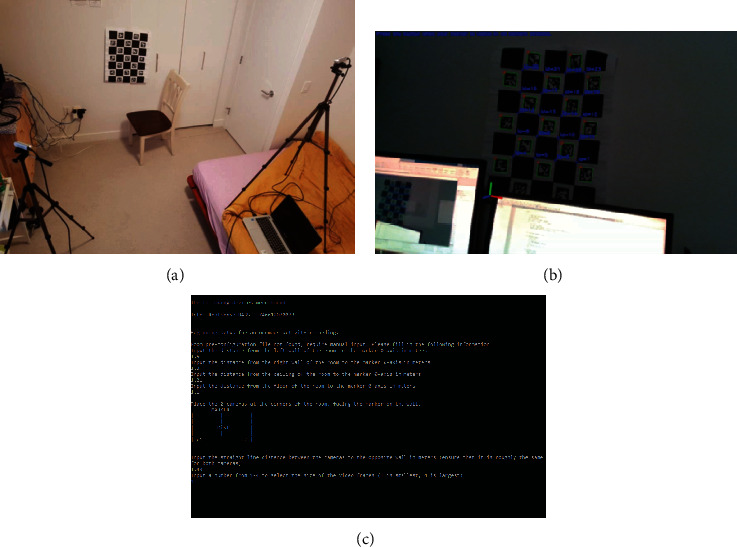
Initialization steps for setting-up the monitoring environment for our study: (a) a typical monitoring area showing the placement of two RGB-D sensors and the calibration prop/object; (b) the resolved world coordinate associated with the monitoring area (red, green, and blue coordinate axes). This coordinate system is then used to merge point clouds obtained from all sensors. (c) Complementary calibration follow-up procedure for associating the physical dimensions of the monitoring scene to the sensed information.

The section presents an integration overview of all major components of the proposed passive monitoring system.  Figure 3(a) shows a view of a typical monitoring environment consisting of two RGB-D sensors, ChArUco calibration prop/object, and a common scene. Initially, both sensors have full to partial view of the calibration prop in the monitoring area. In a typical monitoring application where sensors are placed in any fixed position, the calibration prop can be removed after this initial step.  Figure 3(b) shows an example of identified calibration prop through RGB view of one of the sensors. The image also shows the resolved world coordinate frame located at the bottom left corner of the prop. This coordinate frame is also used to reconstruct the virtual model of the actual scene. In this example, the red, green, and blue colors are used to designate the axes of the corresponding world coordinate frame.  Figure 3(c) shows a follow-up calibration procedure for associating the units of the world coordinates to the physical dimension of the monitoring area (i.e., pixel to the real-world relationships). The setup requires to enter the distances from the walls, ceiling, and floor of the room to the origin of the calibration prop in meters.

**Figure 4:**
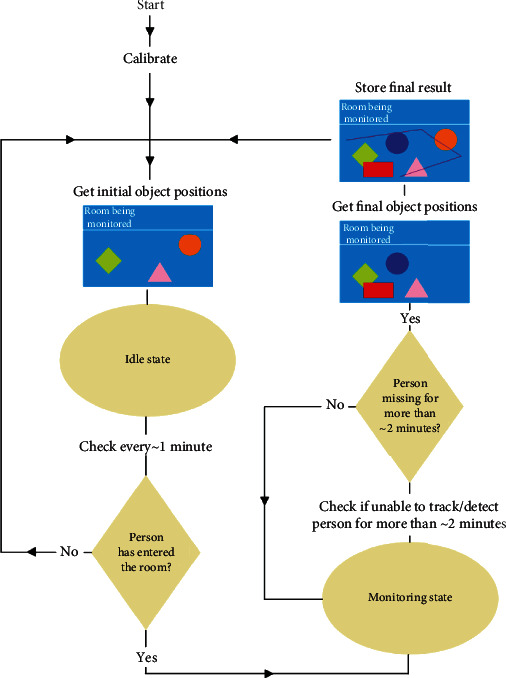
Flow diagram of the proposed passive monitoring system.

Figure 4 shows the overall flow diagram of the proposed passive observer system. As described above, after the initial calibration of the sensor network and at the start of each monitoring cycle, the sensor network captures, detects, classifies, and computes the placement of any known objects in the scene. Using the calibration parameters of the sensor network, it is possible to determine the relative positions of detected objects with respect to each other (this is what is referred to as detection and localization of objects). The proposed object detection and localization system are in an idle state until the presence of the older adult is detected in the scene of the sensor network. During the absence of any movements, the system enters the next cycle of object detection and localization phase until again movements of older adult is detected in the scene.

## 4. Case Studies

We have investigated the performance of the proposed novel integrated system using several monitoring areas associated with a typical living environment of an older adult. In the following, we present two of such studies associated with the living room and the kitchen. The main objective of this study was to demonstrate the feasibility of having a passive observer system which can be utilized as an activity summary that can then be viewed by a family member or caregiving personnel. In order to demonstrate the proof of concept of our implementation, we have used the existing dataset associated with a release version of YOLO implementation.

### 4.1. The Living Room

**Figure 5:**
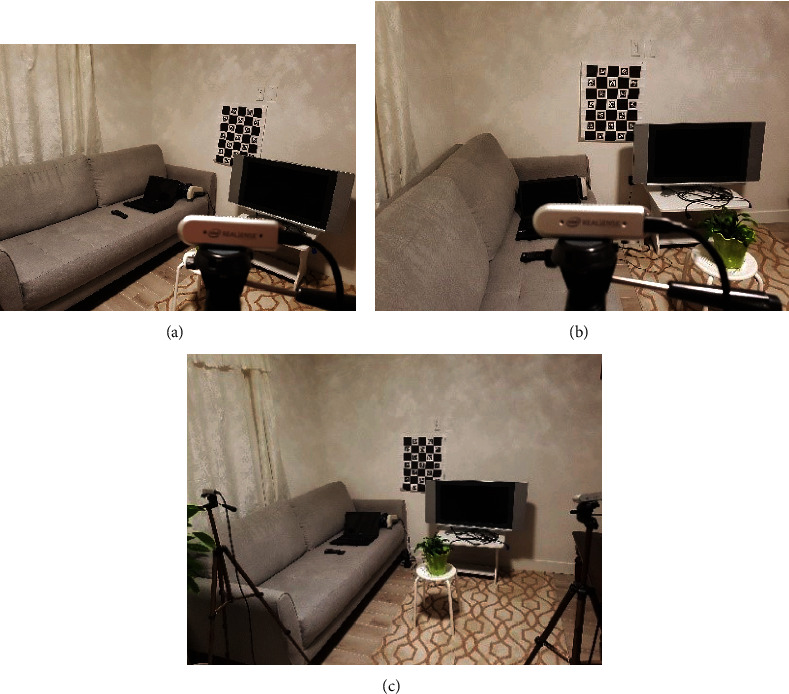
Example of sensor setup for the first case study after the older adult has left the monitoring area. (a, b) show the RGB view from the left and right sensors. (c) shows the full view of the monitoring area including the locations of the left and right sensors.

This case study investigates the detection and localization of objects in a living room environment for before and after the event when an older adult enters the monitoring area (Figure 5).

**Figure 6:**
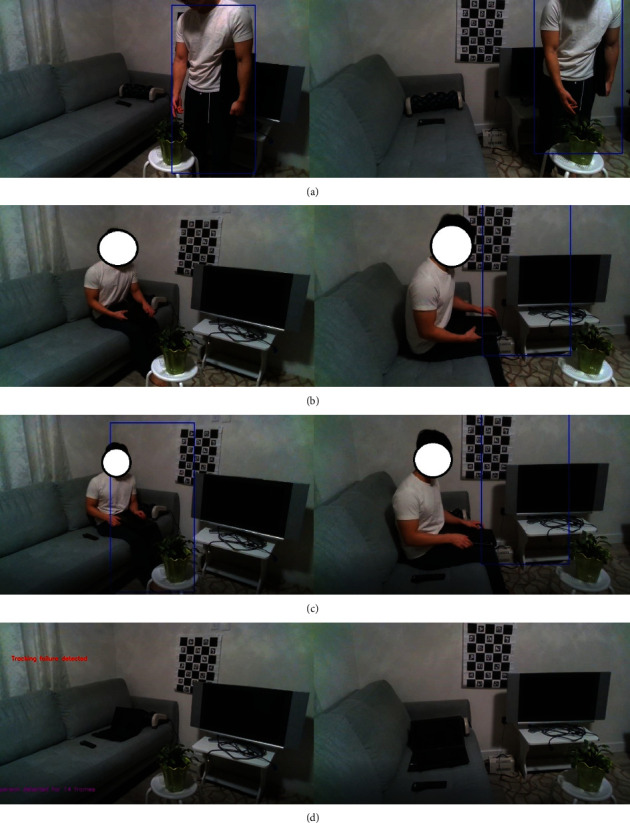
Sequence of activities associated with the first case study when the presence and then the absence of the person are detected in the monitoring area. As a part of the privacy protection feature of the method, the data collection applies when the absence of older adult in the monitoring area is detected. The blue rectangle is the region of interest defined as a part of the movement tracking algorithm.

For example, Figure 6 shows a sequence of screenshots associated with this case study for when the presence of person is detected in the monitoring area.  Figure 6(a) shows when the presence of the person is detected in the living room. The person holding a laptop approaches the *non-italics sofa* where the remote controller for the TV is located (Figure 6(b)). The person then sits down while moving the remote from his left side to his right side (Figure 6(c)). The person opens his laptop then exits the monitoring area while placing the laptop on the sofa (Figure 6(d)).

Following the proposed method, the detected objects are then segmented in the combined calibrated virtual reality point cloud model of the monitoring area. Based on the position and type of detected objects, a simple virtual summary scene is also created using basic geometrical shapes. This simpler scene can then be used as a visual summary by the family member or caregiving personnel for the before and after events.

**Figure 7:**
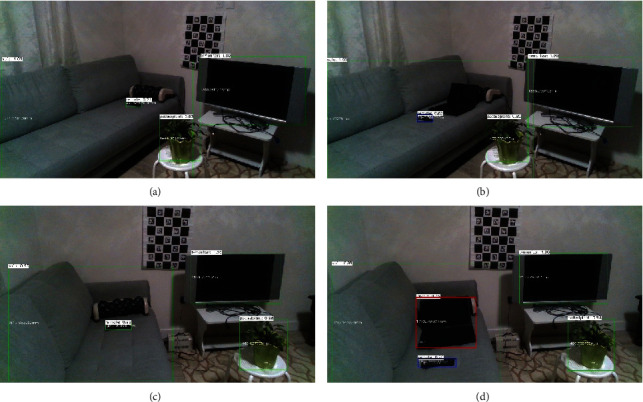
Object detection for before and after the instances when an older adult walks into the monitoring area. Left column (a, c) shows the detected objects at the initial event from left and right sensors, and the right column (b, d) shows the detected objects after the movement event.

As stated, in this case study, the person holding a laptop walks in the monitoring area of the living room. The initial detected objects before presence of the person in the room are sofa, potted plant, TV monitor, and a TV remote controller on the sofa (Figure 6). Figures 7(a) and 7(c) show the initial detected objects in the left and right sensors. The person holding a laptop then walks in the room and stops by a potted plant and then eventually sits on the sofa by moving the TV remote controller further to the middle of the sofa. After a while, the person leaves the monitoring area of the living room by leaving the laptop on the sofa. Figures 7(b) and 7(d) show the detected objects after the older adult leaves the monitoring area.

**Figure 8:**
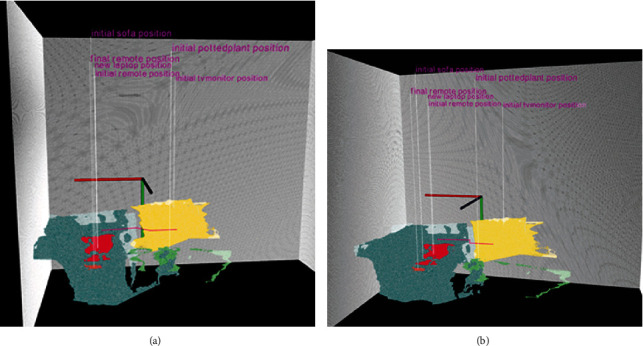
Two views of the calibrated virtual scene based on the merged point clouds of the two depth sensors. Both views show the segmented portion of the point clouds associated with the detected objects after the older adult has left the monitoring area. Virtual reconstruction size of the point cloud is based on the physical sizes of the objects. The detected objects in the RGB image are used to segment the associated point cloud for determining their approximate position with respect to the real and virtual scene. For example, the orange path represents point clouds associated with the final position of the remote. Although it is not used as a part of the results of this paper, pink path shows an example trajectory of person while active in the room.

Figure 8 shows examples of two viewing angles of the calibrated virtual point cloud reconstruction of the living room for when the older adults has left the room. The figure also shows how the detected objects are used to segment the merged point clouds of the two sensors from the monitoring area. The colored segmented point clouds are annotated for better visualization in order to show (a) initial positions of objects before detection of the presence of a person, (b) final positions of objects for in cases where the previously detected objects have been displaced, and (c) presence of any new objects if they were not detected in the initial scene. The figure also shows a simplified trajectory of the movements (it is shown in pink color) of the person between the initial and final detection in the scene (this trajectory information is not used as a part of the analysis of this paper).

**Figure 9:**
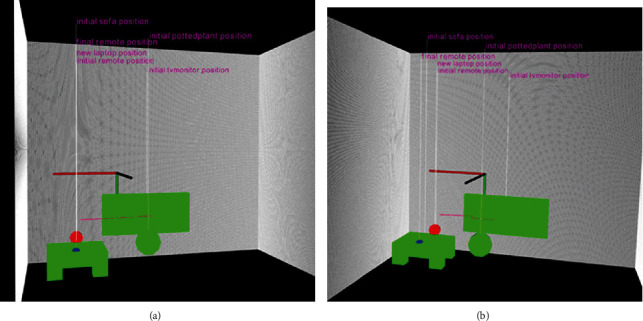
A simplified example of virtual scene summary based on the actual segmented point cloud of Figure 8. This simplified version can further be populated with the actual CAD model of the object for better representation.

Figure 9 shows simplified virtual reality scenes of the monitoring area. This simplified model gives an intuitive representation of the scene where the segmented objects are represented by simple shapes at their actual locations in the real scene. As a part of the visualization experience, representations of objects which have not changed their positions in before and after scenes are shaded in green, and objects whose locations have changed between the scenes are shaded in blue, and objects which were introduced by the older adult in the scene are shown in red.

### 4.2. The Kitchen

**Figure 10:**
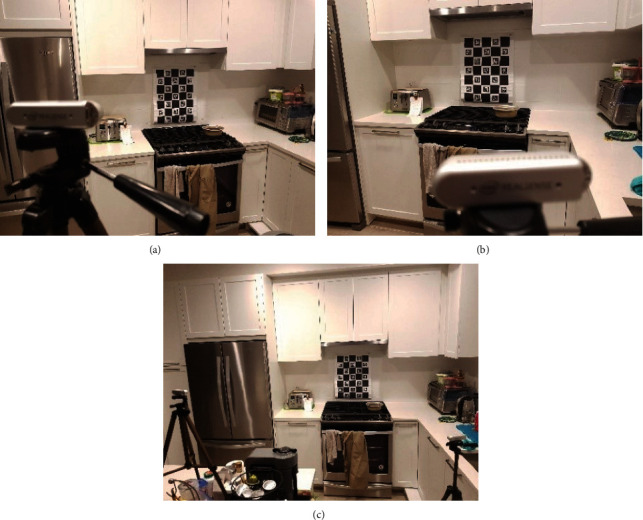
Example of sensor setup for the fourth case study including an oven, a cooking bowl, a toaster, and a microwave oven. (a, b) show views including the left and right sensors. (c) shows the full view of the monitoring area including the locations of the left and right sensor.

**Figure 11:**
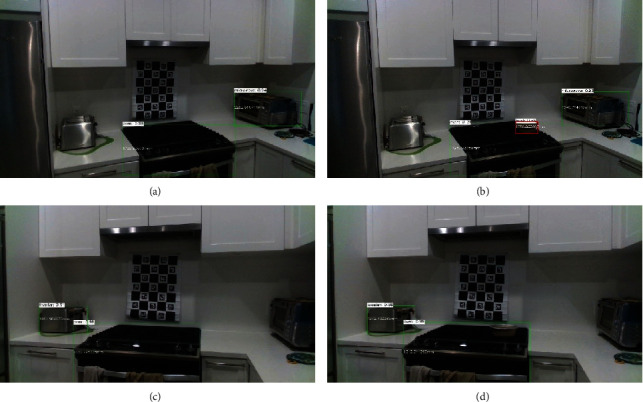
Object detection for before and after the instances when an older adult walks in the kitchen monitoring area and places a cooking bowl on the oven. Left column (a, c) is the detected objects from left (a) and right (c) sensors before an older adult walks into the scene. The right column (b, d) is the detected objects from the left (b) and right (d) sensors when the older adult leaves the scene. Notice the bowl was not detected in the view of the right sensor for when the older adult leaves the room. However, calibration results are used to segment the point cloud in view of the right sensor.

**Figure 12:**
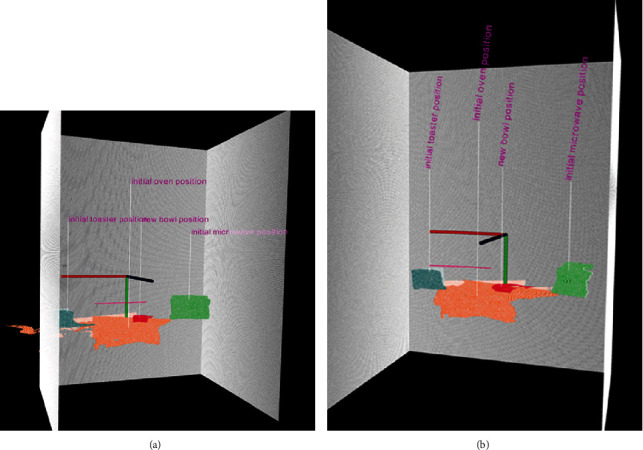
Similar to Figure 8, this figure shows two views of the calibrated virtual scene of the kitchen based on the merged point clouds of the two depth sensors. Both views show the segmented portion of the point clouds associated with the detected objects after the older adult has left the monitoring area. Virtual reconstruction size of the point cloud is based on the physical sizes of the objects. The detected objects in the RGB image are used to segment the associated point cloud for determining their approximate position with respect to the real and virtual scenes. For example, the orange points represent point clouds associated with the final position of the oven. Although the movement trajectory information is not used in this paper, the figure shows also a sample movement trajectory of older adult in the monitoring area.

**Figure 13:**
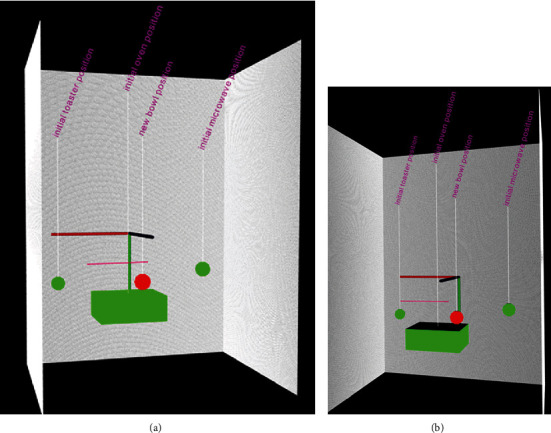
Two views of a simplified example of virtual scene summary based on the actual segmented point cloud of Figure 12. This simplified version can further be populated with the actual CAD model of the object for better representation.

The monitoring area is the view of an oven where the older adult enters the scene and places a bowl on the heating element. Like the previous case studies, Figures 10–13 summarize the detection, localization, and virtual reconstruction of the scene for before and after events. The virtual scenes also show a portion of the movement trajectory of the older adult in the monitoring area.

## 5. Scene Analysis

Previous section demonstrated the feasibility of the proposed passive monitoring system in detecting known objects in the living environment using RGB images from multiple sensors. It also shows how the detected objects from sensors are used in the calibrated point cloud representation using depth information in order to further segment their corresponding positions in the scene. The annotations of the reconstructed scene in defining graphical summary for the before and after events shown, for example, in Figures 9 and 13 are useful for developing visual analytics in association with the movements and activities of older adults. For example, based on locations of objects which an older adult can interact with, various storyboarding can be developed in order to construct their overall activities. The approach is similar to semantic storytelling which has been studied in various other fields [30].

**Figure 14:**
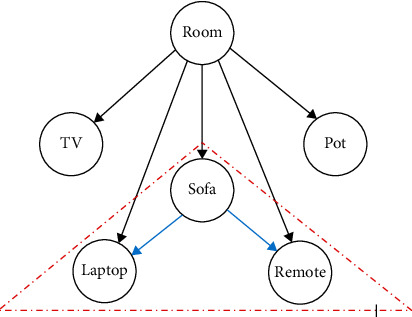
Scene graph representation associated with the first case study. Dark arrows represent the absolute position of the objects with respect to the room coordinate frame system. The colored arrows represent the relative position of the object. Objects in the dashed enclosure represent an example of objects which can be clustered together.

In this paper, we propose an approach based on analysis of the scene graph obtained through the detection and localization of objects in a network of RGB-D sensors [31]. In computer graphics, scene graph is defined as a general data structure which arranges the logical and spatial representations of a graphical scene. For example, in the development of virtual training or gaming environment where the user needs to interact with the graphical objects, it is required to determine the relative positions of the graphical objects with respect to each other in order to determine instances of their contact and interactions [32]. In our study, the information regarding the detected known objects in the graphical scene are obtained from the actual scene. This information is related to the object types and their relative position with respect to the world coordinate system which can be associated with the scene graph. For example, Figure 14 shows an example of such representation associated with the first case study. Scene graphs are collection of nodes in a graph or tree structure connected to normally form a directed acyclic graph (DAG). Nodes are connected by edges which describes the relative position and orientation of the objects with respect to the coordinates of the parent node. In Figure 14, the world coordinate frame defined by the calibration prop/object used to represent a reference for both the physical monitoring area and its virtual model reconstruction. The figure shows the graph of the scene defined by the detected objects in the living room. As stated, this room is represented by 5 different objects which were detected in the initial scene (before detection of movements) based on the RGB information obtained from the sensors. These detected objects are the TV, plant, sofa, laptop, and a remote. Each of the RGB sensors has contributed in the overall detection of these objects through YOLO object detection implementation. The detected objects are segmented in the merged point cloud of the depth sensors where the information regarding their spatial locations of their representative points in the segmented point clouds are computed. In general, edges of the scene graph represent the position and orientation of the reference coordinate of the graphical objects with respect to the parent nodes. In this initial study, we utilized the relative position (distances) between the corresponding nodes.

Referring to Figure 14, the dark-colored directed edges represent the position of the detected objects with respect to the calibration world coordinate frame. Similar to Figure 1, the direction of arrow represents the relative position of an object designated by the head of the arrow with respect to the frame of an object defined by the tail of the arrow. The colored arrows represent the relative position of an object with respect to each other which can be computed using the absolute descriptions of position. For example, since both the remote and laptop are on the sofa, the positional descriptions of colored arrows can be used to cluster the sofa, laptop, and remote together. The main idea of the proposed scene analysis is to determine if any of the detected objects have been displaced or removed between the before and after events. This can be accomplished by computing the Euclidean distance of each of the object with respect to the World reference frame. In an event where the before and after distances of the same detected object is less than a predefined threshold, it can be concluded that the object has not been moved. In contrary, if the difference in the distance to the origin of the world coordinate is greater than some predefined value, it can be stated that the object is moved by the older adult. In case there are differences in nodal description of the scene graph for before and after detection of the events, i.e., by comparing the graph structure, it can be assumed that a new object was either added or taken away between the events of older adult entering and leaving the monitoring area.

Tables 1 and 2 show the coordinates of the center points associated with the segmented point clouds of each of the detected objects associated with the above case studies. Following a basic distance computation, it is possible to identify objects which are handled by older adults between each of the monitoring cycles. It was also shown that by a simple comparison of the scene graphs representation for before and after events, it is also possible to determine if there is any new object added or taken away from the monitoring area.

As it can be seen from the position estimation of objects for before and after events in case study one (Table 1) and the distance computation, it can be seen that the remote is moved, and the laptop is added. For case study two and comparing the position of the objects for before and after events, it can be seen that the bowl is added to the scene during this monitoring cycle.

## 6. Conclusions

Supporting independent living of lifestyle of active older adults can be beneficial in enhancing their sense of self-reliance on providing themselves with basic needs and may also offer them a better financial alternative. To support this option, a comprehensive design needed to be in place in order to make such choice a viable alternative to various assisted or community living environment. Monitoring movements and activities can be one of the main components of such a system which can play a central role in support of the independent living lifestyle. One of the main requirements of this deployment is the level of detail taken to protect the privacy. This paper proposes an approach which can be utilized as a part of monitoring system. It consists of a network of calibrated RGB-D sensors which can be deployed at various living spaces of older adults. As an option, the system can be only activated when the older adult is present in the monitoring area, and only the placement of objects can be used to further infer the expected activities. This way, no information about the older adult can be collected. The system can be trained to detect various objects which are associated with the living environment of the older adult. Using physical dimensions, the system can be reconstructed in a virtual scene based on the monitoring space having the exact representations of the placement of the objects. Using the scene graph analysis, the system can further infer if any of the objects has been displaced by the older adults while being in the monitoring area or if any objects has been introduced or taken away from the scene. A simplified annotated virtual scene can be used by caregivers or family members in order to give some indications regarding possible activities of older adults. Combined with other passive information which can be obtained through smart home network, it can be possible to create a complete storyboard of movements and activities of older adults without disclosing any visual information that can be considered as personal.

The case studies of this paper assumed that event data for when the older adult is not present in the scene can be collected from various unrelated scenes or other passive sensors (for demonstration of this paper, we have used visual tracking algorithms for obtaining spatial trajectory of motion). Also, only information regarding some global taxonomy of motion of objects or objects with respect to each other is used. For example, from the scene graph representation of the virtual reconstruction, one can further infer about causal relationships between the objects. For example, in Figure 14, groups of objects can be associated together to either belonging to the same vicinity or have some causal relationships such as one object being on top of the other (this is shown within the red triangle where both the remote and laptop can be inferred to be on top of the sofa). The overall accuracy of the system is dependent on many factors. These can be the illumination condition of the monitoring area, the accuracy of the object detection, and the segmentation of the point cloud for creation of visual summary. For example, in case of the presence of identical objects in the living space, a decision-making algorithm needs to be further implemented in order to distinguish between them. The method of this paper implemented two sensors in order to resolve any occlusion issues. However, if there will be cases where objects can be occluded from the view of both sensors, a third or more additional sensors need to be deployed in the monitoring area.

The method of this paper can further be expanded to monitoring kids or children of young age in indoor environments. Depending on the level of detail needed, various monitoring areas in the living space can be enhanced where more details regarding postures and modes of interaction of the older adult with the objects can be collected [33]. Examples of such setup would be the top view of the dining room table where a network of calibrated RGB-D sensors is placed. The setup can be used as a part of the stroke rehabilitation for monitoring grasping and manipulation during dining. The segmented point cloud of the objects can further be refined with their CAD models in order to increase the level of detail on how and where the objects are grasped.

## Figures and Tables

**Table 1:** Spatial position of the detected objects with respect to the living room coordinate frame.

Object	Initial position (*x*, *y*, *z*) cm	Final position (*x*, *y*, *z*) cm
Remote	(67, 93, 46)	(71, 96, 94)
Potted plant	(-23, 86, 100)	(-22, 85, 98)
Sofa	(70, 84, 79)	(69, 83, 77)
TV	(-42, 61, 24)	(-42, 60, 23)
Laptop		(67, 88, 48)

**Table 2:** Spatial position of the detected objects in the kitchen coordinate frame.

Object	Initial position (*x*, *y*, *z*) cm	Final position (*x*, *y*, *z*) cm
Microwave	(-82, 43, 41)	(-83, 45, 40)
Oven	(17, 67, 59)	(15, 65, 61)
Toaster	(93, 58, 17)	(91, 56, 16)
Bowl		(-5, 57, 40)

## Data Availability

Associated data with the segmented and reconstructed scenes is available from the authors.
